# Memory effect behavior with respect to the crystal grain size in the organic-inorganic hybrid perovskite nonvolatile resistive random access memory

**DOI:** 10.1038/s41598-017-16805-4

**Published:** 2017-11-29

**Authors:** Jin Hyuck Heo, Dong Hee Shin, Sang Hwa Moon, Min Ho Lee, Do Hun Kim, Seol Hee Oh, William Jo, Sang Hyuk Im

**Affiliations:** 1 Department of Chemical and Biological Engineering, Korea University, 145 Anam-ro, Seongbuk-gu, Seoul 136-713 Republic of Korea; 2 Department of Physics, Ewha Womans University, 52 Ewhayeodae-gil, Seodaemun-gu, Seoul, 03760 Republic of Korea

## Abstract

The crystal grain size of CH_3_NH_3_PbI_3_ (MAPbI_3_) organic-inorganic hybrid perovskite (OHP) film was controllable in the range from ~60 nm to ~600 nm by non-solvents inter-diffusion controlled crystallization process in dripping crystallization method for the formation of perovskite film. The MAPbI_3_ OHP non-volatile resistive random access memory with ~60 nm crystal grain size exhibited >0.1 TB/in^2^ storage capacity, >600 cycles endurance, >10^4^ s data retention time, ~0.7 V set, and ~−0.61 V re-set bias voltage.

## Introduction

Currently the memory devices are the essential compartments of most electronic devices in our daily life. Most of all, the resistive random access memory (ReRAM) has been considered as promising next-generation nonvolatile memory device due to its simple device architecture, high memory density, fast operation speed, and low power consumption. The ReRAM can handle the information by resistive switching (RS) effect originated from the conductive filament formation^1^, the crystalline-amorphous phase transition^2,3^, and the charge storaging/trapping^4,5^.

Recently, organic-inorganic hybrid perovskite (OHP) materials have received a lot of attention as great potential material for various applications, such as thin-film transistors^6–8^, detectors^9–11^, light-emitting diodes (LEDs)^12–14^, and solar cells^15–17^ due to their high absorption coefficient, convenient band gap tailoring, long diffusion length of charge carriers, ambipolar charge transport, and solution processability at low/mild temperature. Especially, they exhibited excellent performance in the solar cells so intensive studies have been done to find commercial applications. Although the record efficiency of organic-inorganic hybrid perovskite solar cells have been achieved over 20%^17^, it was known that the perovskite hybrid solar cells often exhibited significant hysteresis of photocurrent density-voltage (*J-V*) curves with respect to scan rate and direction due to displacement current of ferroelectric materials with multi-domain structures, ions/defects migration by ionic crystalline characteristics of perovskite material, and charge trapping/detrapping in bulk and interface of perovskite material^18–21^. This implies that the hysteresis will be dependent on the domain size of perovskite film. Namely, it is expected that the smaller crystalline domain will exhibit the larger hysteresis which improve the memory performance.

Very recently, Yoo *et al*. reported low temperature processable CH_3_NH_3_PbI_3−x_Cl_x_ (MAPbI_3−x_Cl_x_) mixed halide perovskite based ReRAM device comprised of FTO (F dope SnO_2_)/MAPbI_3−x_Cl_x_/Au^22^, and RRAM device using Ag active electrode instead of Au inert electrode^23^, respectively. Gu *et al*.^24^ reported MAPbI_3_ perovskite based flexible ReRAM comprised of PET/ITO (indium tin oxide)/MAPbI_3_/Au. Muthu *et al*.^25^ reported MAPbBr_3−x_Cl_x_ mixed halide perovskite nanoparticles based ReRAM device comprised of FTO/MAPbBr_3−x_Cl_x_/Ag. (see the summary of performance in Table S1).

Although few reports have been disclosed, there are no reports on the effect of crystal grain size on the performance of OHP-ReRAM. To make poly crystal MAPbI_3_ OHP film, here, we adapted the dripping process^26^ because the dripped non-solvent induces immediate nucleation/solidification of the OHP film, whereas the solubility controlled crystallization process^27,28^ makes quasi-single crystalline OHP film. We controlled the crystal grain size of MAPbI_3_ OHP film by non-solvents inter-diffusion controlled crystallization in dripping process which uses the toluene and iso-propyl alcohol (IPA) non-solvent mixture as a dripping solvent. In addition, we systematically studied the effect of crystal grain size of MAPbI_3_ OHP film on the performance of OHP-ReRAM.

## Results

### Formation of MAPbI_3_ OHP film by non-solvent inter-diffusion controlled dripping process. 

**Figure 1:**
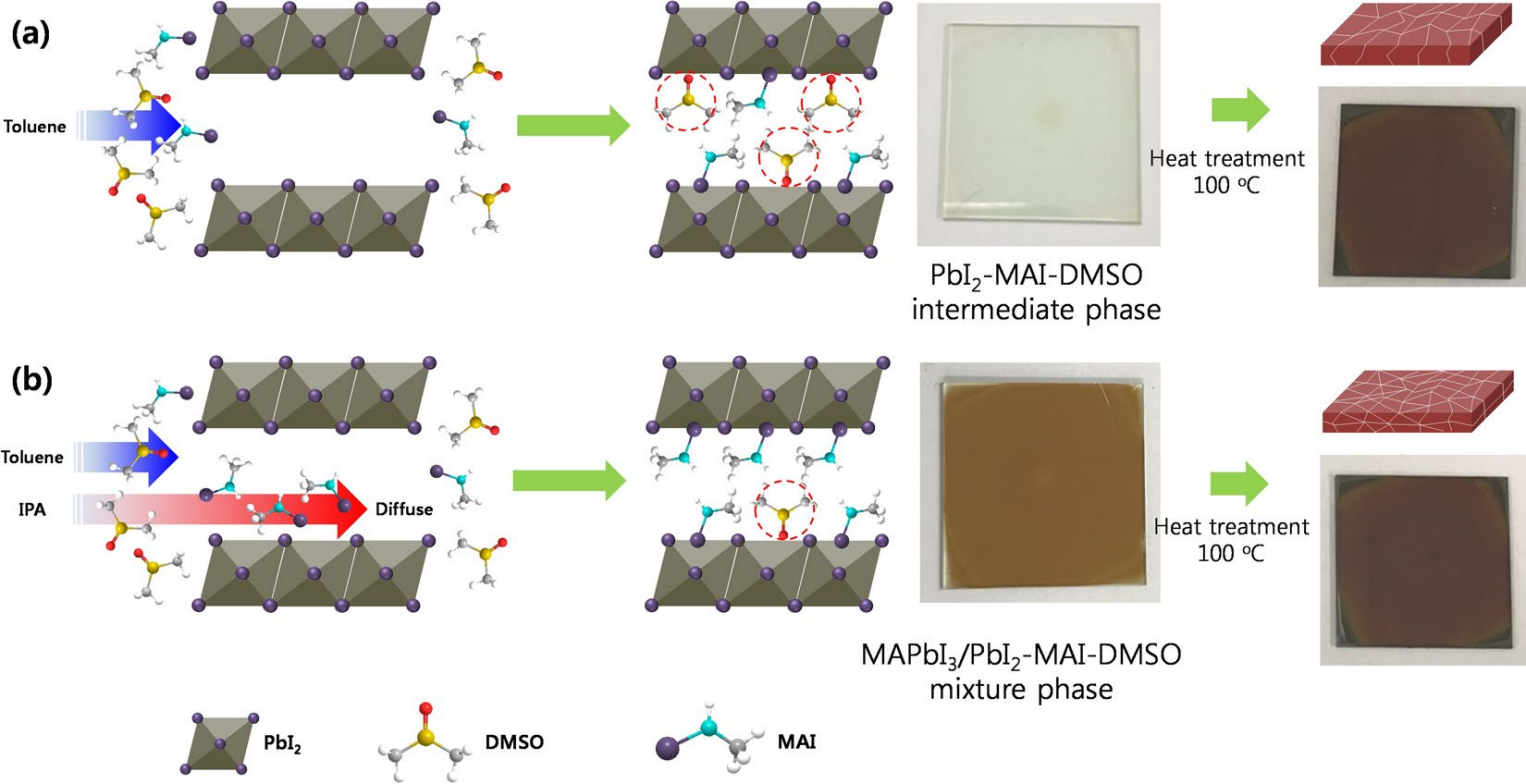
Schematic illustration for the formation of MAPbI_3_ OHP film with controlled crystal grain size by conventional non-solvent dripping process (**a**) and non-solvent inter-diffusion controlled dripping process (**b**).

Figure 1 shows a schematic illustration for formation of MAPbI_3_ OHP film with controlled crystal grain size by conventional non-solvent dripping process (Fig. 1(a)) and non-solvent inter-diffusion controlled dripping process (Fig. 1(b)). When forming the OHP film by spin-coating process, the uniform wet-film is formed on the substrate by spin-off of excess solution. At the moment, the liquid wet-film can be abruptly transformed into the solid PbI_2_-MAI-DMSO (dimethyl sulfoxide) intermediate film by the dripping non-solvent. The conventional non-solvent dripping process forms the PbI_2_-MAI-DMSO intermediate phase by toluene dripping during spin-coating of MAPbI_3_/GBL (γ-butyrolactone)/DMSO solution because toluene does not dissolve the MAPbI_3_ OHP film and is miscible with DMSO so that toluene can induce PbI_2_-MAI-DMSO intermediate phase and removes the excess DMSO except for DMSO involving the formation of the intermediate phase. Accordingly, a transparent PbI_2_-MAI-DMSO intermediate film is formed by toluene dripping process as shown in Fig. 1(a). Additional heat-treatment at 100 °C for 2 min is necessary to convert the PbI_2_-MAI-DMSO intermediate phase into dark brown MAPbI_3_ OHP film (see Fig. 1(a)) because DMSO adduct can be easily released from the intermediate phase by heat-treatment. On the other hands, the non-solvent inter-diffusion controlled dripping process uses a toluene and IPA non-solvent mixture to make smaller MAPbI_3_ crystal grains because the IPA (solubility parameter non-solvent (δ) = 23.5 MPa^0.5^) is more miscible to DMSO (δ = 26.7 MPa^0.5^) than the toluene (δ = 18.2 MPa^0.5^) non-solvent and consequently it can directly form the MAPbI_3_ OHP film by dripping process as shown in Fig. 1(b). In other words, the toluene and IPA non-solvent mixture can more quickly transform the wet-film into the solid phase MAPbI_3_ film while the PbI_2_-MAI-DMSO is partially remained in the film. The transformation ratio of MAPbI_3_ and PbI_2_-MAI-DMSO intermediate phase by the dripping of the toluene and IPA non-solvent mixture will be dependent on the concentration of IPA. To fully convert the remained PbI_2_-MAI-DMSO intermediate phase, additional heat-treatment is also required as shown in Fig. 1(b). Accordingly, the smaller MAPbI_3_ OHP grains will be formed by dripping of the toluene and IPA non-solvent mixture because the IPA non-solvent more miscible to DMSO is more quickly inter-diffused into the wet-film and consequently the more number of nuclei is created.

**Figure 2:**
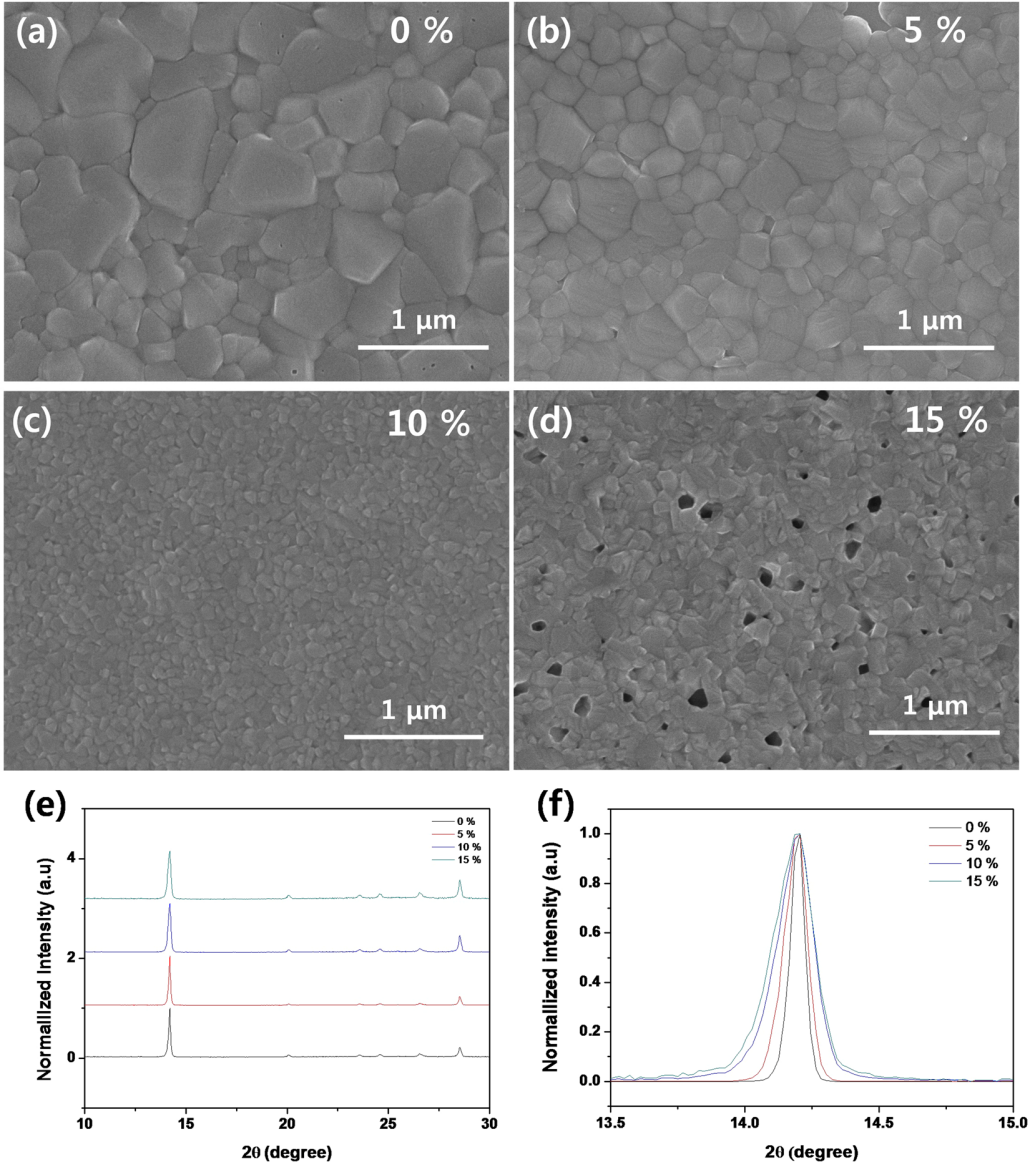
SEM (scanning electron microscopy) surface images of the resulted MAPbI_3_ OHP film on the ITO substrate with respect to the different concentration of IPA non-solvent in the IPA/toluene dripping solvent: (**a**) 0%, (**b**) 5%, (**c**) 10%, and (**d**) 15 volume % and their corresponding XRD (X-ray diffraction) patterns (**e**,**f**): f is magnified XRD peak of (110) in Fig. 2(e).

Figure 2(a–d) are the SEM (scanning electron microscopy) surface images of the resulted MAPbI_3_ OHP film on ITO substrate with respect to the different concentration of IPA non-solvent (0%, 5%, 10%, and 15 volume %) in the dripping toluene and IPA mixture solvent. The SEM image of the MAPbI_3_ OHP film clearly indicates that the grain size of MAPbI_3_ perovskite crystal dramatically decreased and saturated with the volume ratio of IPA from 0 to 15 in the dripping solvent. The average diameter of the crystal grains was ~600 nm for the 0% sample, ~200 nm for the 5% sample, ~60 nm for the 10%, and ~70 nm for 15% sample, respectively as summarized in Table 1. However, the pin-holes were formed in the 15% sample due to the abrupt shrinkage of the wet-film by extraction of a large amount of DMSO at a time. To confirm the crystal structure and crystal grain size of MAPbI_3_ OHP film by the concentration of IPA in the dripping solvent, we checked XRD (X-ray diffraction) patterns as shown in Fig. 2(e). The XRD patterns did not show any impurity peaks, such as PbI_2_, indicating the formation of a pure perovskite phase. To compare the average crystal grain size of MAPbI_3_ OHP film, we calculated the average crystal grain size by using the Scherrer equation^29^.

1 $${\rm{t}}=0.9\lambda \cdot {(\mathrm{Bcos}{\rm{\theta }})}^{-1}$$

where *t* is the average crystallite size, *λ* is the wavelength of the X-ray irradiation (0.154 nm), and *B* is the line width at half maximum (in radians). To calculate the average crystal grain size of the MAPbI_3_ OHP film, we used the (110) peak at 2θ = 14.21°, and obtained the calculated average crystal grain size of the MAPbI_3_ OHP film to ~220 nm for the 0% sample, ~120 nm for the 5% sample, ~65 nm for the 10% sample, and ~70 nm for the 15% sample in Fig. 2(f). Accordingly, we obtained the uniform MAPbI_3_ OHP film with the smallest grain size via non-solvent inter-diffusion controlled dripping process.

**Table 1:** Summary of grain sizes, trap density, and set and re-set voltage of MAPbI_3_ OHP ReRAM with controlled crystal grain size.

Solvent composition (IPA vol. %)	Average diameter of crystal grains^a^ (nm)	Average crystallite size^b^ (nm)	Set voltage (V)	Re-set Voltage (V)	Trap density^c^ (cm^−3^)
0	600	220	0.66	−0.58	2.91 × 10^15^
5	200	120	0.68	−0.59	3.00 × 10^15^
10	60	65	0.70	−0.61	3.13 × 10^15^
15	70	70	0.70	−0.61	3.17 × 10^15^

^a^Obtained from SEM images in Fig. 2(a–d), ^b^calculated from equation 1, ^c^calculated from equation 2.

### Memory properties of MAPbI_3_ OHP ReRAM with controlled grain size

**Figure 3:**
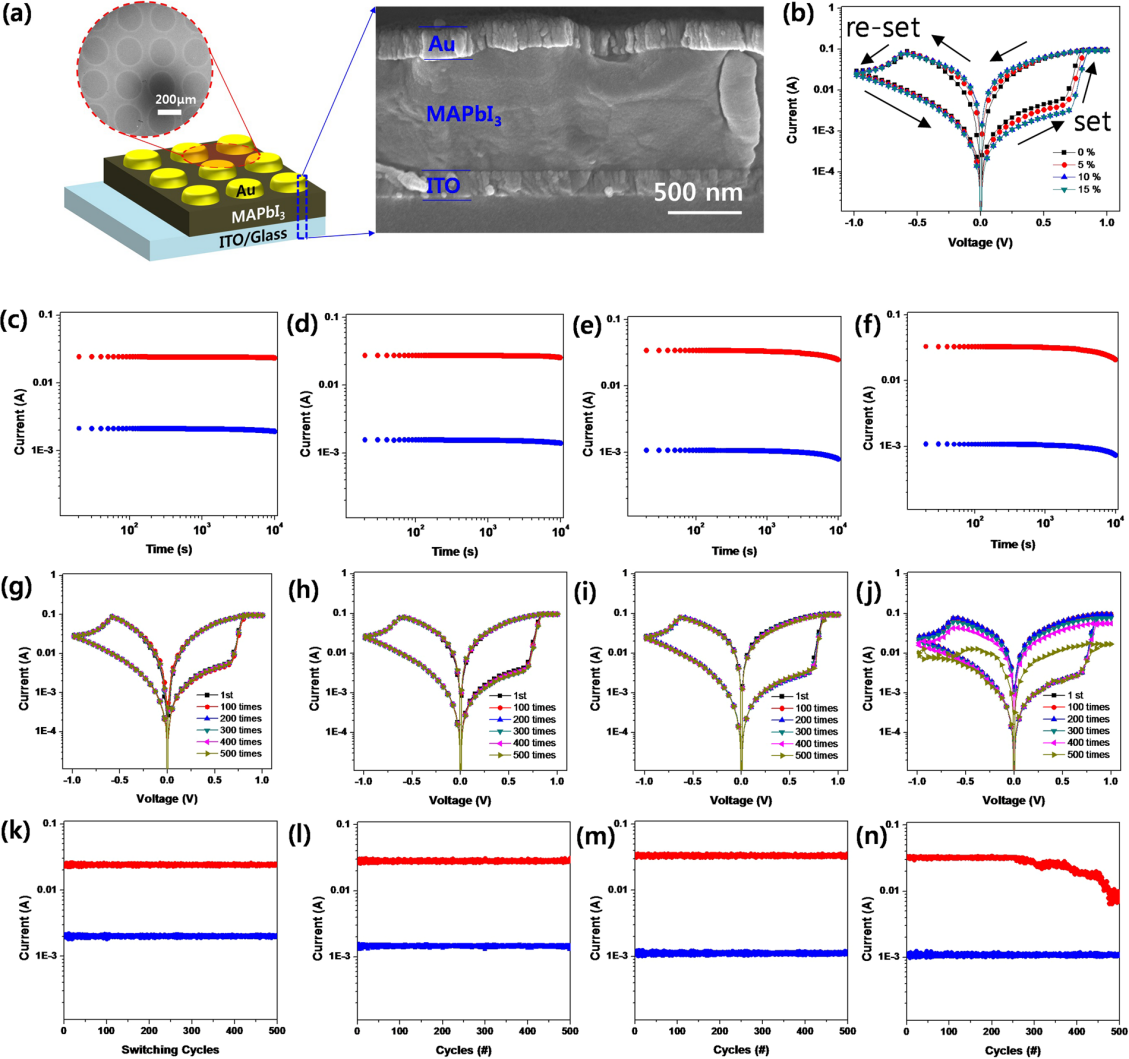
(**a**) A schematic illustration of ReRAM device structure consisting of ITO/MAPbI_3_ OHP film with controlled grain size/Au and the SEM (scanning electron microscopy) images of real device: SEM top-surface image = the circular Au top electrode arrays with 250 nm in diameter and 50 nm pitch formed on the MAPbI_3_ OHP film, SEM cross-sectional image = cross-sectional image of the ReRAM; (**b**) I-V curves of the MAPbI_3_ OHP ReRAM devices with respect to the crystal grain size under sweeping a DC bias voltage (0 V → 1 V → 0 V → −1 V → 0 V); and (**c**–**f**) high-resistance state (HRS: red) and low-resistance state (LRS:blue) with retention time, (**g**–**j**) I-V curves by the repeated number of cycles, and (**k**–**n**) endurance of HRS (red) and LRS (blue) at 0.25 V_ro_ of MAPbI_3_ OHP ReRAM prepared by dripping 0% (**c**,**g**,**k**), 5% (**d**,**h**,**l**), 10% (**e**,**i**,**m**), and 15% (**f**,**j**,**n**) IPA non-solvent.

To check the effect of the crystal grain size in MAPbI_3_ OHP film on the RS property, we fabricated ReRAM devices with the MAPbI_3_ OHP film with different grain size. Figure 3(a) shows a schematic illustration of ReRAM device structure consisting of ITO/MAPbI_3_ OHP film with controlled grain size/Au. The SEM (scanning electron microscopy) top-surface image shows that the circular Au top electrode array with 250 nm in diameter and 50 nm pitch is formed on the MAPbI_3_ OHP film with controlled grain size. The SEM cross-sectional image shows that the thickness of ITO, MAPbI_3_ OHP film, and Au layer is ~100 nm, ~800 nm, and ~110 nm, respectively. Figure 3(b) is I-V characteristics of the MAPbI_3_ OHP ReRAM devices with respect to the crystal grain size under sweeping a DC bias voltage (0 V → 1 V → 0 V → −1 V → 0 V). Under sweeping the DC bias voltage from 0 V to 1 V, the set process (writing), which is the switching of resistance state from high-resistance state (HRS) to low-resistance state (LRS), was occurred at ~0.66 V for 0%, ~0.68 V for 5%, ~0.7 V for 10%, and ~0.7 V for 15% sample, respectively. The current level was also lowered and saturated before reaching on setting voltage as the grain size is reduced. This clearly shows that the set process depends on the crystal grain size of MAPbI_3_ OHP film. Under sweeping DC bias voltage from 0 V to −1 V, the re-set process (deleting), which is the switching of resistance state form LRS to HRS, was occurred at ~−0.58 V, ~−0.59 V, ~−0.61 V, and ~−0.61 V, respectively. Accordingly, the LRS/HRS ratio of ReRAM devices is gradually increased and saturated as the crystal grain size of MAPbI_3_ OHP film reduced and the set and re-set voltages are slightly increased. Although the MAPbI_3_ OHP ReRAM devices show bipolar RS characteristics, we intentionally choose the 0.25 V as read out voltage (V_ro_) which exhibits the highest the ratio of LRS/HRS. To confirm the electrical reliability of ReRAM devices, we measured the HRS and LRS values at 0.25 V_ro_ as shown in Fig. 3(c–f). The HRS and LRS values were very slightly degraded over long time, but the HRS/LRS ratio was almost constant up to 10^4^ s.

To check the endurance of the MAPbI_3_ OHP ReRAM devices with respect to the crystal grain size, we repeatedly measured their I-V characteristic for 500 cycles and recorded the HRS and LRS values at 0.25 V_ro_ for 600 cycles as shown in Fig. 3(g–n). Except for 15% sample, all of the MAPbI_3_ OHP ReRAM devices showed same I-V curves even after 500 cycles. The degradation of MAPbI_3_ OHP ReRAM device of 15% sample might be attributed to the non-uniform MAPbI_3_ OHP film because the Au metal electrode cannot be uniformed deposited on the pin-holes in the non-uniform film. The AFM (atomic force microscopy) topology images in Fig. S1 indicate that the 10 and 15% sample has rms roughness (R_q_) of 4.8 and 14.3 nm, respectively. This might cause the uncertainty of applied bias voltage in the film and degradation of perovskite material as well due to the weak passivation by Au electrode under working experimental condition. From above experimental results, we can rationally conclude that the MAPbI_3_ OHP non-volatile ReRAM with controlled crystal grain size will have good endurance if uniform MAPbI_3_ OHP film is made.

**Figure 4:**
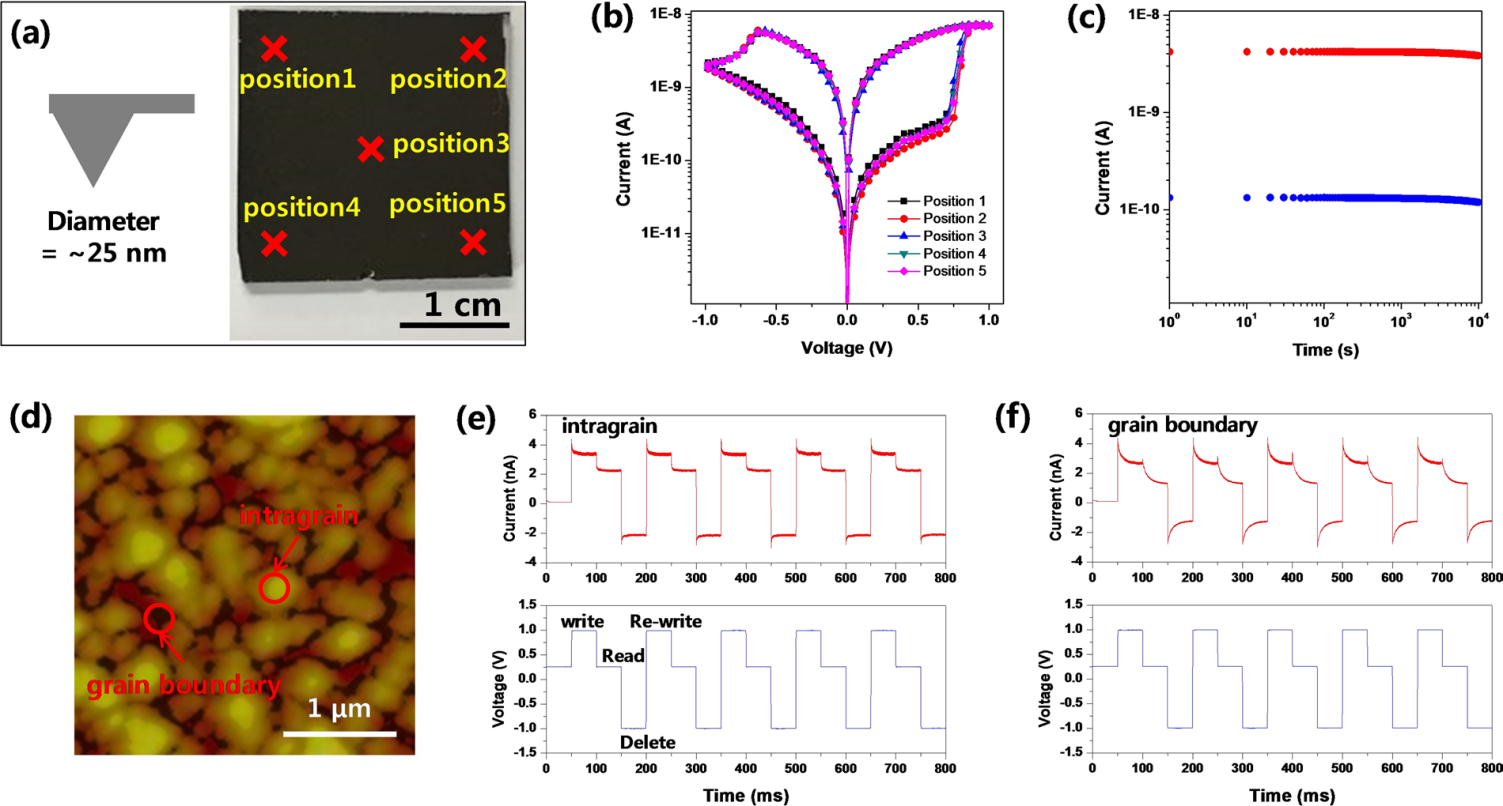
(**a**) Photograph of MAPbI_3_ OHP film with 2.54 cm × 2.54 cm in area and schematic illustration of the tip for conductive AFM (atomic force microscopy): the five red ×  = measured positions of I-V curves, (**b**) corresponding I-V curves of 5 marked positions in Fig. 4(a), (**c**) HRS (red) and LRS (blue) at 0.25 V_ro_ with retention time, (**d**) CAFM image of MAPbI_3_ film with Cr/Au coated tip biases of 1 V, (**e**) the response current signals at the intragrain with respect to the repeated write/read/delelet/re-write process: upper signal = response current, lower signal = applied bias voltage, (**c**) the same signals for the grain boundary.

In order to check the uniformity and storage capacity of the MAPbI_3_ OHP ReRAM, we re-checked the RS effect by using conductive AFM (cAFM: atomic force microscopy) tip. The diameter of Au/Cr AFM tip was ~25 nm and we measured the RS effect on the 5 different positions as shown in Fig. 4(a). The I-V characteristics at 5 different positions of the MAPbI_3_ OHP ReRAM film prepared with 10% IPA non-solvent dripping solution was shown in Fig. 4(b). These results clearly indicate that the RS effects of 5 different positions are almost identical so we can conclude that the uniformity of the MAPbI_3_ OHP film is quite good. The set process (writing) of HRS to LRS transition was occurred at ~0.7 V and the re-set process (deleting) of LRS to HRS transition was done −0.61 V, respectively. We also checked stable reading process between the set and the re-set operation by applying smaller bias voltage into the device. The HRS/LRS ratio was maintained over 10^4^ s at 0.25 V_ro_ as shown in Fig. 4(c). We measured the cAFM image of MAPbI_3_ perovskite OHP film with Cr/Au coated tip biases of 1 V as shown in Fig. 4(d). We also performed the cAFM inside of the grains and near the grain boundaries, and as a result, point-by-point measurements of cAFM shows the distinct current flow behaviors, as shown in Fig. 4(e,f). Even though the contacts between the tip and the surface are the exactly same, the repeated measurements are likely excluding the contact problems with nice measured current characteristics in Fig. 4(e,f). For the 50 msec-pulse of the external bias voltage, the response current in the intragrains exhibits quite a stable and constant characteristics. After the ‘write’ process, the ‘read’ is successfully detected. And, the ‘delete’ and ‘re-write’ processes can clearly recover the same conductions state. Also, the MAPbI_3_ OHP ReRAM device exhibited fast operation speed without significant time delay. These mean that the MAPbI_3_ perovskite behaves as memristor and the device can record information quickly. However, the response in the grain boundary looks transient and decreasing quickly. The whole responses are similar to the intragrains but too leaky to keep the charges.

**Figure 5:**
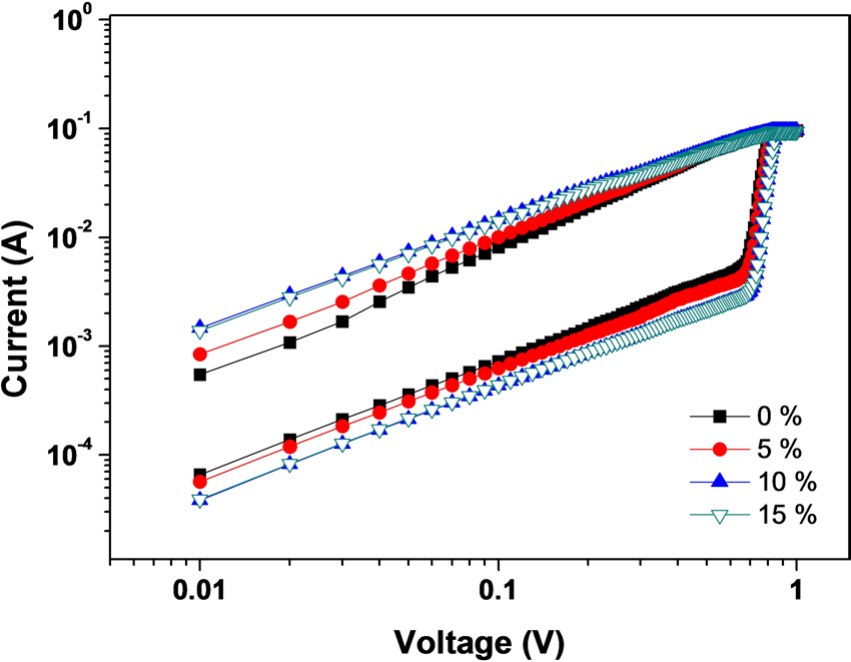
Current density vs. bias voltage curves of MAPbI_3_ OHP ReRAM with different concentration of IPA non-solvent.

We measured trap densities at high voltage region in order to understand why the RS effect is slightly dependent on the crystal grain size of the MAPbI_3_ OHP non-volatile ReRAM as shown in Fig. 5. All devices exhibited the ohmic behavior at low voltage and the trap-filled-limited (TFL) transport at high voltage. The voltage value for the transition (V_TFL_) is dependent on the trap density of the device as following^30,31^:

2 $${{\rm{V}}}_{{\rm{TFL}}}={{\rm{eN}}}_{{\rm{t}}}{{\rm{d}}}^{2}\cdot {(2{{\rm{\varepsilon }}{\rm{\varepsilon }}}_{{\rm{0}}})}^{-1}$$

where e, d, ε, ε_0_, N_t_ is elementary charge, thickness of MAPbI_3_ OHP film, and dielectric constant in vacuum, and trap density, respectively. The caluclated trap density (N_t_) of each sample was ~2.91 × 10^15^ cm^−3^, ~3.00 × 10^15^ cm^−3^, ~3.13 × 10^15^ cm^−3^, and ~3.17 × 10^15^ cm^−3^, respectively as summarized Table 1. The slightly increased trap density with respect to the reduced crystal grain size of MAPbI_3_ OHP film indicates that the traps are originated by surface traps in the grain boundaries.

To better understand the MAPbI_3_ OHP ReRAM, we measured the polarization vs. bias voltage curves with the different concentration of IPA non-solvent using probe station (PS1 Series, MODUSYSTEMS) combined with ferroelectric tester (Precision LC II, RADIANT) at 1 kHz at room temperature in the dark as shown in Fig. S2. These results indicated that MAPbI_3_ OHP ReRAM was leaky dielectric behavior rather than ferroelectric characteristics and their values were greatly dependent on the crystal grain size. It is interesting that the leaky relaxation increases as the crystal grain size reduces, but we cannot understand exactly why it is happened in current situation. So further studies are need to understand the underlying physics of the MAPbI_3_ OHP ReRAM with controlled crystal grain size.

## Disucssion

In summary, we could control the crystal grain size of MAPbI_3_ OHP film in the range from ~60 nm to ~600 nm by the non-solvents inter-diffusion controlled crystallization process. The crystal grain size was controllable by dripping the toluene and IPA non-solvent mixture with controlled IPA concentration on the perovskite wet film because the IPA (solubility parameter non-solvent (δ) = 23.5 MPa^0.5^) non-solvent more miscible to DMSO (δ = 26.7 MPa^0.5^) than the toluene (δ = 18.2 MPa^0.5^) can more quickly transform the wet-film into the solid phase MAPbI_3_ film. As a result, we could fabricate MAPbI_3_ OHP film with ~60 nm in grain size by dripping 10% IPA non-solvent. The RS effect was dependent on the crystal grain size of MAPbI_3_ OHP ReRAM and eventually, the 10% sample exhibited the highest performance. As a results, the MAPbI_3_ OHP ReRAM with ~60 nm crystal grain size (10% sample) exhibited >0.1 TB/in^2^ storage capacity, > 600 cycles endurance, >10^4^ s data retention time, ~0.7 V set, and ~−0.61 V re-set bias voltage. In addition, there was no significant delay to set and re-set the information. From the cAFM analysis of the intra-crystal grains (grain inside) and inter-crystal grains (grain boundary), the memory behaviors are well maintained in intra-crystal grains but are not in inter-crystal grains. Here, we found that the film uniformity and crystal grain size affect the performance of memory effect, but it is not clearly understood what is the exact origins to make RS effect in polycrystalline perovskite film because the trap densities are not greatly changed with crystal grain sizes and leaky dielectric behavior is dependent on the crystal grain size. So further studies will be required to understand the origins of RS effect in the perovskite films. However, we believe that the perovskite based ReRAM device with controlled crystal grain size will be promising candidate for next-generation ReRAM.

## Methods

### Preparation of 1 M MAPbI_3_ perovskite solution

Methylamine (35 mL, 40% in water, TCI) and hydriodic acid (50 mL, 57 wt% in water, Aldrich) were reacted in a 250 mL round-bottom flask at 0 °C for 2 h under magnetic stirring. The precipitate was recovered by evaporation at 50 °C for 1 h. The product was then dissolved in ethanol (anhydrous 99.9%, Samchun pure chemical co., ltd.) recrystallized from diethyl ether (99.0%, Samchun pure chemical co., ltd.) and finally dried at room temperature in a vacuum oven for 24 h. 1 M of MAPbI_3_ perovskite solution was prepared by mixing the synthesized MAI powder (0.318 g) and PbI_2_ (0.924 g, 99%, Aldrich) in a mixture of γ-butyrolactone (1.4 mL, Aldrich) and dimethyl sulfoxide (0.6 mL, Aldrich) at 60 °C for 30 min.

### Device fabrication

ITO glass (10 Ω sq^−1^, AMG) was cleaned by ultrasonication with ethanol (anhydrous 99.9%, Samchun pure chemical co., ltd.) for 15 min, and then treated Ar plasma for 2 min. MAPbI_3_ was coated on the ITO glass by two-step spin coating process at 1000 and 2000 r.p.m for 10 and 30 s, respectively. Just before it increased from 1000 to 2000 r.p.m, 1 mL of toluene (anhydrous 99.8%, Aldrich) and IPA (anhydrous 99.5%, Aldrich) mixture was quickly dropped onto the center of the substrate. The substrate was dried on a hot plate at 100 °C for 5 min. Finally, an Au top electrode was deposited by thermal evaporation. Au top electrode arrays was 250 μm in diameter and 50 μm pitch.

### Device characterization

Morphological images of the surface and cross section were taken by high resolution field emission scanning microscopy (HR FE-SEM, Merlin, Carl Zeiss) with 10 kV acceleration voltage. The crystal structure and crystal grin size were analyzed by X-ray diffraction (XRD, D8 Advance, Bruker) with Cu Kα radiation at a step size of 0.02°. The current-voltage (I-V) characteristics of devices were measured by potentiostat (Ivium Stat, Ivium) with probe station (PS1 Series, Modu systems). The scan rate of I-V measurements was fixed at 10 mV/200 ms. The uniformity and time response of device were obtained by measuring conductive atomic force microscope (C-AFM, XE-100, park system).

## Electronic supplementary material

Supplementary Information
