# Supplementary material for: Memory effect behavior with respect to the crystal grain size in the organic-inorganic hybrid perovskite nonvolatile resistive random access memory
Source: Sci Rep. 2017 Nov 29;7:16586. doi: 10.1038/s41598-017-16805-4 (PMC5707385; doi:10.1038/s41598-017-16805-4)
Supplement: Supplementary file 1 — Supplementary Information [file 41598_2017_16805_MOESM1_ESM.pdf]

Supplementary information

Memory effect behavior with respect to the crystal grain size in the organic-inorganic hybrid perovskite nonvolatile resistive random access memory

Jin Hyuck Heo, [1] Dong Hee Shin, [1] Sang Hwa Moon, [1] Min Ho Lee, [1] Do Hun Kim, [1] Seol Hee Oh,[2] William Jo,[2] and Sang Hyuk Im*[1]

[1] Department of Chemical and Biological Engineering, Korea University, 145 Anam-ro, Seongbuk-gu, Seoul 136-713, Republic of Korea

[2] Department of Physics, Ewa Womans University, 52 Ewhayeodae-gil, Seodaemun-gu, Seoul 03760, Republic of Korea

* To whom correspondence should be addressed. E-mail: imromy@korea.ac.kr

Table S1. Summary of the performance of organic-inorganic perovskite material based memory devices.

Material	Device structure	Set voltage	On/off ratio	Retention time	Ref#
$CH_3NH_3PbI_{3-x}Cl_x$	Glass/FTO/$CH_3NH_3PbI_3$/Au	0.8 V	~10^1 <	~10^4 s	20
$CH_3NH_3PbI_{3-x}Cl_x$	Glass/FTO/$CH_3NH_3PbI_3$/Au	1.4 V	~10^2 <	~10^4 s	21
$CH_3NH_3PbI_3$	PET/ITO/$CH_3NH_3PbI_3$/Au	0.7 V	~10^2 <	~10^4 s	22
$CH_3NH_3PbBr_{3-x}Cl_x$ (x=0, 0.46, 1.03) nanoparticles	Glass/FTO/$CH_3NH_3PbBr_{3-x}Cl_x$/Ag	2.66 V/1.04 V/0.53 V	~10^1 <	~10^3 s	23

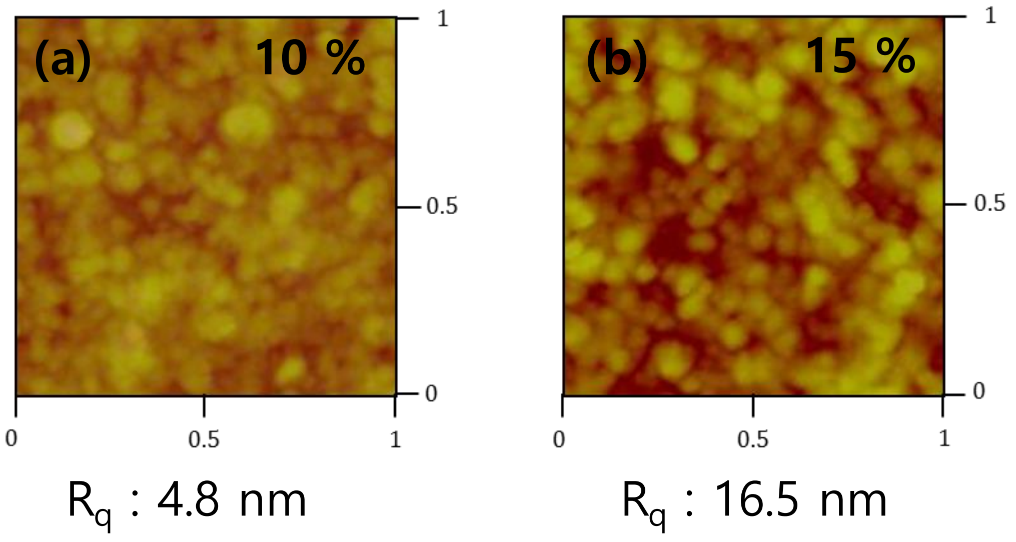

Fig. S1. AFM topology of (a) 10 % and (b) 20 % sample (R_q = rms roughness).

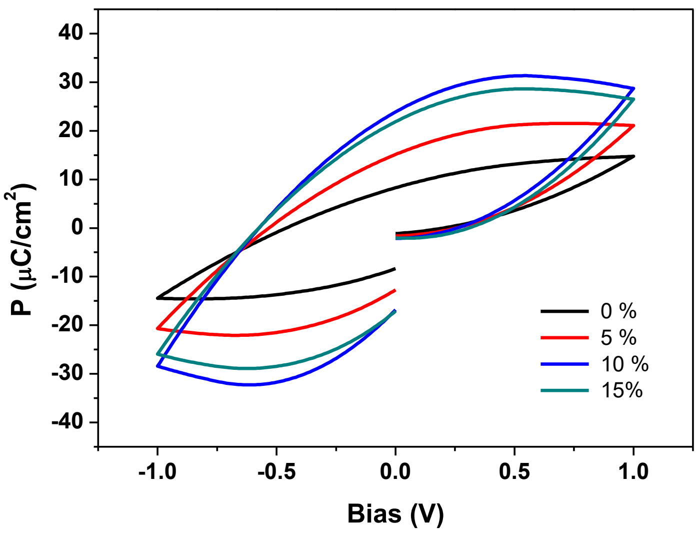

Fig. S2. Polarization vs. bias voltage curves with different concentration of IPA non-solvent.
